# Facial signals shape predictions about the nature of upcoming conversational responses

**DOI:** 10.1038/s41598-025-85192-y

**Published:** 2025-01-09

**Authors:** Alexandra K. Emmendorfer, Judith Holler

**Affiliations:** 1 Donders Institute for Brain, Cognition and Behaviour, Radboud University, Wundtlaan 1, 6525 XD Nijmegen, Nijmegen, The Netherlands; 2 Max Planck Institute for Psycholinguistics, Nijmegen, The Netherlands

**Keywords:** Social interaction, Facial signals, Eye gaze, Preference organization, (Dis)affiliative responses, Language, Psychology, Human behaviour

## Abstract

Increasing evidence suggests that interlocutors use visual communicative signals to form predictions about unfolding utterances, but there is little data on the predictive potential of facial signals in conversation. In an online experiment with virtual agents, we examine whether facial signals produced by an addressee may allow speakers to anticipate the response to a question *before* it is given. Participants (*n* = 80) viewed videos of short conversation fragments between two virtual humans. Each fragment ended with the Questioner asking a question, followed by a pause during which the Responder looked either straight at the Questioner (baseline), or averted their gaze, or accompanied the straight gaze with one of the following facial signals: brow raise, brow frown, nose wrinkle, smile, squint, mouth corner pulled back (dimpler). Participants then indicated on a 6-point scale whether they expected a “yes” or “no” response. Analyses revealed that all signals received different ratings relative to the baseline: brow raises, dimplers, and smiles were associated with more positive responses, gaze aversions, brow frowns, nose wrinkles, and squints with more negative responses. Qur findings show that interlocutors may form strong associations between facial signals and upcoming responses to questions, highlighting their predictive potential in face-to-face conversation.

**Supplementary Information:**

The online version contains supplementary material available at 10.1038/s41598-025-85192-y.

## Introduction

In conversation, interlocutors take turns at speaking, and these turns often form socially contingent actions^1–3^. For example, a speaker might invite an addressee to their party, to which the addressee may respond by accepting or declining the invitation. Conversation analysis describes this type of exchange as an adjacency pair^4,5^. Here, the invitation is considered an initiating action, while accepting or declining the invitation is a responding action. Responding actions can further be described as “preferred” or “dispreferred”, where preferred responses are often positive, or affiliative in nature (e.g., complying with a request), and dispreferred responses often negative, or disaffiliative (e.g., declining an invitation when the speaker of the invitation might have expected a positive response)^6^. In conversation analysis, the field of study investigating how preferred and dispreferred actions naturally occur and are organized in social interaction is called preference organization.

Evidence from conversational corpora suggests that interlocutors may be able to anticipate whether an addressee is about to give a preferred or dispreferred response based on both verbal and non-verbal cues. Dispreferred responses may be preceded by particles (e.g., *uh*,* well*), or simply a longer pause, while preferred responses are more often immediate and direct^7^. More specifically, while typical gap durations for responses to questions lie around 200 ms, responses given after 700 ms are more likely to be dispreferred^7^. EEG studies suggest that interlocutors are indeed sensitive to this type of gap duration and response preference in conversation, and use gap length to form expectations about the upcoming response^8,9^. Forming such expectations is useful for the producer of an initiating actions since it may allow them to react with mitigating actions in case the expected response is disaffiliative^7,10^. In other words, receiving early predictive signals may allow speakers of initiating actions to rephrase their utterance such that an affiliative response becomes more likely (see^10^, p. 20). For example, an invitation to a party (‘Do you want to come to my party on Friday?’), upon receiving signals foreshadowing a disaffiliative response (such as ‘Oh sorry, no, I can’t’), may be reworded in such a way that increases the likelihood of an affiliative response by allowing the response to conform with the expectations suggested by the speaker’s turn design (e.g. ‘I suppose you’re busy at the moment and won’t be able to come to my party on Friday? -- Response: Indeed, I can’t, sorry). While the core information provided in the response is the same, the latter corresponds with the speaker’s implied expectation. This results in a less face-threatening response, and would be considered preferred over the former. Alternatively, in case speakers of an initiating action do not opt for rephrasing the utterance, perceiving early signals predictive of the type of response that is about to follow would still allow them to know early on how to react to that upcoming response in their next turn (e.g. ‘Oh that’s a shame’, where a fast response might sound more sincere than a delayed one). Thus, perceiving early signals produced by a next speaker may facilitate predictions by the current speaker, allowing them to either quickly rephrase what they just said, or to prepare a swift response to the responding turn which the early signals are foreshadowing.

In addition to the gap duration preceding a response, visual communicative signals may offer an additional early predictive cue to the nature of the upcoming response. While psycholinguistic research has classically focused on speech, in its natural environment, human language is a multimodal phenomenon^11–15^. Recent frameworks propose that the concurrent visual signals produced by interlocutors in face-to-face conversation shape how language is processed^16^. One mechanism by which visual communicative signals are thought to contribute to language processing is prediction. This is supported by a growing body of evidence: speech sounds can be anticipated from mouth movements^17^, hand gestures may facilitate predictions of semantic information^18^, and both facial signals and hand gestures can be associated with particular social actions^19,20^, and since they occur early during utterances^18,21^, may facilitate also pragmatic prediction.

Much of the research on the ‘predictive potential’^18^ of visual signals has focused on signals produced by the *speaker* during an ongoing verbal utterance. However, interlocutors produce visual signals not only during their verbal turn, but also while their conversation partner is speaking. Such addressee signals may indicate that they are attending to the speaker and are able to follow along^22–24^, but can also occur before repair initiations to signal when there is misunderstanding^25^. Moreover, qualitative research has shown that visual signals produced by the *next* speaker can foreshadow some aspects of the turn they are about to deliver, such as dealing with affiliative challenges or announcing a change in emotional stance^10,26,27^. In the current experiment, we examine whether observers of such visual signals can indeed use them to anticipate upcoming responses before they are given, such as they seem to be able to do with the length of pauses. Specifically, we investigate whether certain visual signals are perceived as projecting more affiliative/preferred (‘yes’) or disaffiliative/dispreferred (‘no’) responses. Gaze is a particularly salient visual communicative signal that can carry social meaning^28–30^. Observations in conversational corpora have already suggested that an addressee’s gaze direction may be associated with whether their upcoming response is a preferred response, such as accepting an invitation, or a dispreferred response, such as rejecting an offer^10^. Interlocutors seem sensitive to this association, as indicated by examples showing that current speakers may use gaze direction to anticipate an upcoming response and adapt their utterances on the fly, in response to perceiving the averted gaze (ibid.). However, while conversational corpora preserve the rich, interactional nature of conversation, they do not allow for conclusions about causal relations. The present experiment thus aims to gain insight into the perception of facial signals and their predictive potential regarding next speaker’s actions. While there is already some evidence linking gaze and response preference, little is known about how other facial communicative signals are linked to response preference. This study also seeks to gain insight into the predictive potential of facial signals more widely. For this purpose, we selected a range of visual facial signals that occur frequently in face-to-face conversation, including in responses to questions^21,26,27,31^.

The current experiment uses a multimodal overhearer paradigm based on virtually animated characters^32^. We select virtually animated characters over video recordings of real humans in order to keep a tight experimental control over the visual facial signals produced. We present participants with short dialogue excerpts ending in a question, where the addressee produces one of eight facial signals (straight gaze, gaze aversion, brow raise, brow frown, nose wrinkle, dimpler, smile, squint), and ask them to rate on a 6-point scale whether they expect a ‘yes’ or a ‘no’ response (ranging from ‘maybe yes’, to ‘probably yes’, to ‘definitely yes’, and their no-equivalents). If interlocutors are sensitive to the association between gaze aversion and response preference, we expect participants’ ratings to be more negative (i.e., more ‘no’ ratings) when the addressee averts their gaze, compared to a baseline condition of straight gaze (the behavior addressees engage in most while listening to questions)^30,33^. Additionally, we explore whether any of the other visual signals presented here are associated with response preference. We hypothesize brow frowns, nose wrinkles and dimplers to be associated with more negative responses compared to the baseline condition, due to their association with the ‘not-face’, a facial expression that can include these facial signals as its Action Unit components in contexts of negation and negative moral judgement^31^. Smiles on the other hand are hypothesized to be linked to more positive (‘yes’) responses due to their generally affiliative nature^26^. Brow raises and squints may be more ambiguous signals: brow raises may be associated with different attitudes such as skepticism or information seeking, depending on the context^34^, while squints might indicate thinking or uncertainty^35^. The present study makes an important step forward by shedding light on how facial signals can shape conversational expectations. Exploratory analyses of empathy scores also provide a first glimpse of how personal social predispositions may modulate this influence.

## Methods

This study was preregistered at https://aspredicted.org/NQF_W3F, approved by the Ethics Committee Social Sciences (ECSW-2018-135) and carried out in accordance with the Declaration of Helsinki. All participants gave their informed consent to participate in the experiment. Data and code are available at https://osf.io/5ehcm/.

### Participants

80 participants completed the online experiment via Prolific and were compensated 11.25 GBP (~ 13€) for their participation. Participants were eligible to participate if they met the following inclusion criteria: aged 18–45, first and primary language reported as Dutch, no hearing difficulties, vision normal or corrected-to-normal, no language-related disorders including dyslexia, currently residing in the Netherlands. Two participants were excluded as they did not meet the inclusion criterion of native Dutch speaker (incorrect inclusion criteria in Prolific, later corrected. These participants reported Dutch as a primary language, but this was learned later in life). One participant was excluded due to failure to perform the task according to the preregistered criteria (66.7% accuracy on comprehension question, and an unusually high number of repeated ratings, see Supplementary Figure S1). The final sample analyzed here consists of 77 participants (39 female, mean age: 27.6 range: 18–45).

### Stimuli

**Fig. 1:**
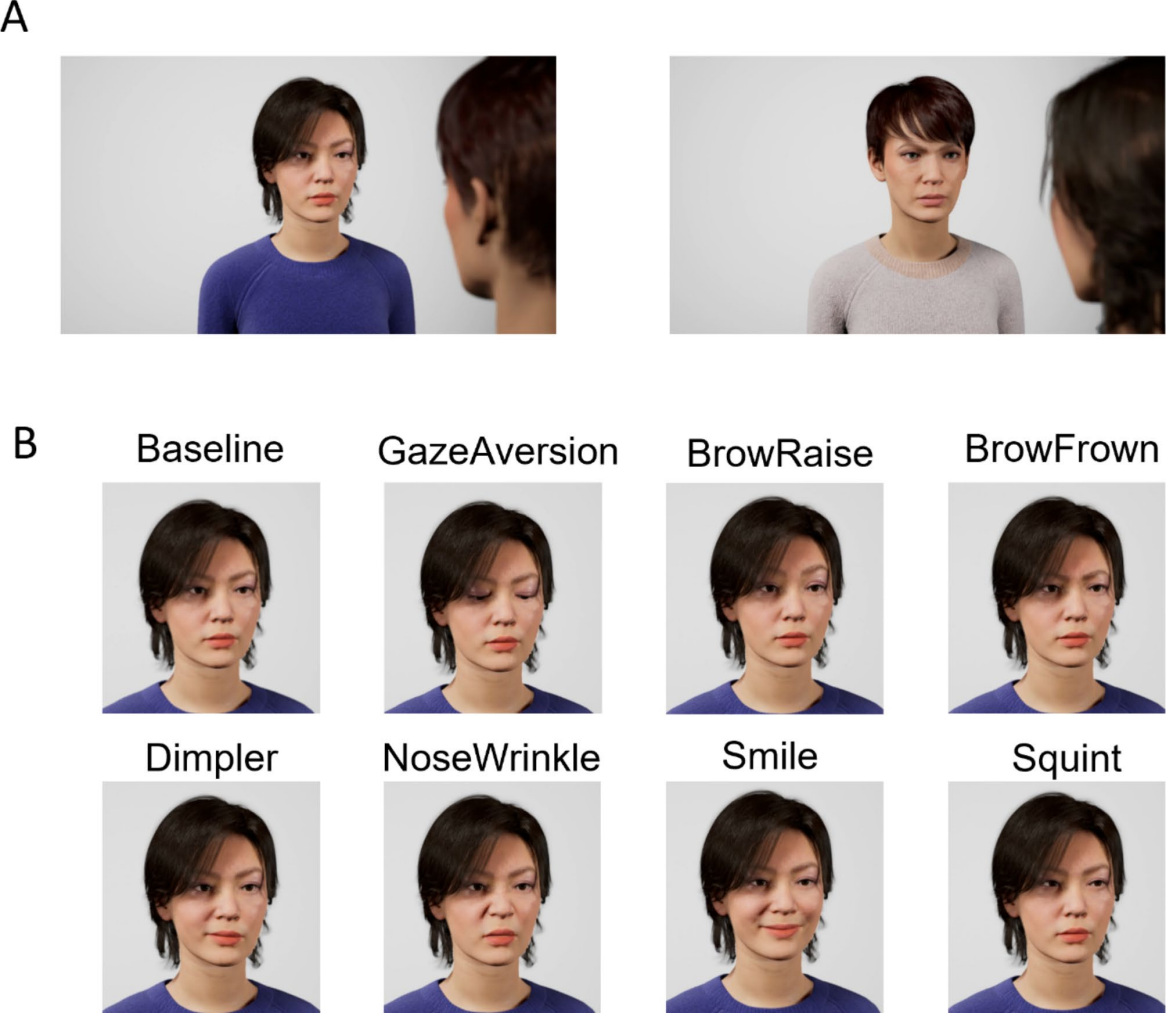
Stimuli. **A** Participants view the scene in an “over-the-shoulder perspective”, where they are looking over the shoulder of the Questioner, at the Responder at the center of the screen. **B** At the end of the question, the Responder is presented in one of 8 visual conditions.

Stimuli consisted of animated videos of two female virtual humans (Questioner and Responder) in face-to-face conversation. Participants viewed the interaction from an over-the-shoulder-perspective of the Questioner, with the Responder’s face at the center of the screen (Fig. 1a). This perspective was intended to make participants feel somewhat immersed despite being observers of the interaction (i.e. creating a third-person-perspective paradigm from as much of a second-person-perspective as possible, as this can affect cognition^36^). The interactions were presented as excerpts of conversations on the topics planning a joint party, vacation, or dinner, moving, or studies. Each excerpt consisted of a brief conversational exchange, ending in a question, which could be either a proposal, offer, invitation or request. The dialogues were spoken by two native Dutch speakers, who were instructed to speak the scripted dialogues in a natural, conversational tone. The speakers were seated in two sound-attenuated booths and could hear each other via headphones. The dialogues were recorded in Audacity at 44.1 kHz 32 bit, and later extracted and edited in Praat (version 6.2.10). This editing only entailed equalizing loudness to 65 dB and a Hann bandpass filter of 80–10500 Hz to remove background noise; no changes were made to the timing of the turns in the recording.

Each interaction was presented in one of 8 visual conditions (Fig. 1b), where the Responder performs one of the following facial signals: straight gaze (baseline condition), downwards gaze aversion, brow raise, brow frown, dimpler, nose wrinkle, smile, or squint. Following a similar animation procedure as reported by Trujillo and Holler^34^, virtual humans were generated using MetaHuman^37^, with facial signals animated in Maya^38^, and synchronized lip-movements automatically generated for each dialogue in Jali (version 1.31)^39^. Additionally, to create a more natural and lifelike animation, some ambient motion including random blinks, ambient saccades and an idle sway were added. Audio, lip-movement, visual signals and idle motion were combined and rendered in Unreal Engine (v.4.27)^37^. The video ended with 24 still frames (1 s) with the visual signal at its final position. After these still frames, the video was replaced by a 6-point Likert-type scale where participants indicated which response they expected.

### Procedure

Participants were asked to attend to the videos, and then indicate on a 6-point Likert-type scale which response they expected the Responder to give (levels: *zeker ja/*definitely yes, *waarschijnlijk ja*/probably yes, *misschien ja*/maybe yes, *misschien nee*/maybe no, *waarschijnlijk nee/*probably no, *zeker nee/*definitely no). After 8 practice trials (1 per visual condition, presented in random order), the main task started. Participants completed 12 blocks with 19–20 videos per block. To monitor attention, 20% of trials were followed by a comprehension question (”Waar ging de dialog over?”/ “What was the dialogue about?”), where participants selected the correct answer from four alternatives. For a sample dialogue and comprehension question, see Table 1. For both the expected response ratings and the comprehension questions participants were given 10 s to respond to ensure they gave a rating based on their immediate impression, and stayed engaged in the online experiment. Mean accuracy on the comprehension questions was 97.0%. =.

After completing the main task, participants completed two questionnaires: the Empathy Quotient (EQ)^40^, and a short questionnaire about their perception of the avatar (Supplementary Figure S2). The EQ ranges from 0 to 80. The median EQ score in the current dataset was 36, ranging from 16 to 75. Avatars were rated for their humanness (median = 4, std. dev = 1.17), ease of understanding (median = 5, std. dev = 0.94), and likeability (median = 4, std.dev = 1.10) on a 6-point scale^19^. The distribution of scores across participants is presented in Supplementary Figure S2.

**Table 1:** Example of an interaction and the corresponding comprehension question.

	Speaker	Transcript	Translation
Context	B	Ik kan mijn notities van het vierde college niet vinden	I can’t find my notes from the fourth lecture
	A	Dat was die over leesontwikkeling he?	That was the one about reading development, right?
Question	A	Wil je die mijne overschrijven?	Do you want to copy mine?
Comprehension question			
		Waar ging de dialog over?	What was the dialogue about?
Correct response		College notities	Lecture notes
Alternatives		Een presentatie Een studieboek Collegebanken	A presentation A textbook Lecture hall seats

#### Statistical analysis

To test whether the facial signals led to different expected responses, we used a cumulative link mixed-effects model using the clmm function of the package ordinal^41^ in R^42^, which is suitable for analyzing ordinal outcome variables such as our 6-point scale. We fit a model with Response as the outcome variable (levels: definitely yes, probably yes, maybe yes, maybe no, probably no, definitely no), visual signal as a fixed effect (levels: straight gaze – baseline, gaze aversion, brow raise, brow frown, dimpler, nosewrinkle, smile, squint), and random intercepts for participant and item (models with randoms slopes did not reach convergence). Using the anova function, this model was compared to a null model containing only random intercepts, to assess whether visual signals improved model fit. Each facial signal was subsequently compared to the reference level of straight gaze (“baseline”), and to each other with emmeans (p-values adjusted with Bonferroni correction). The analysis was repeated using all 80 participants who completed the study, and the results remained the same (see Supplementary Tables S2 and S3). We further explored whether ratings are influenced by participants’ EQ scores (For further details on the analysis, see Supplementary Materials).

## Results

**Fig. 2:**
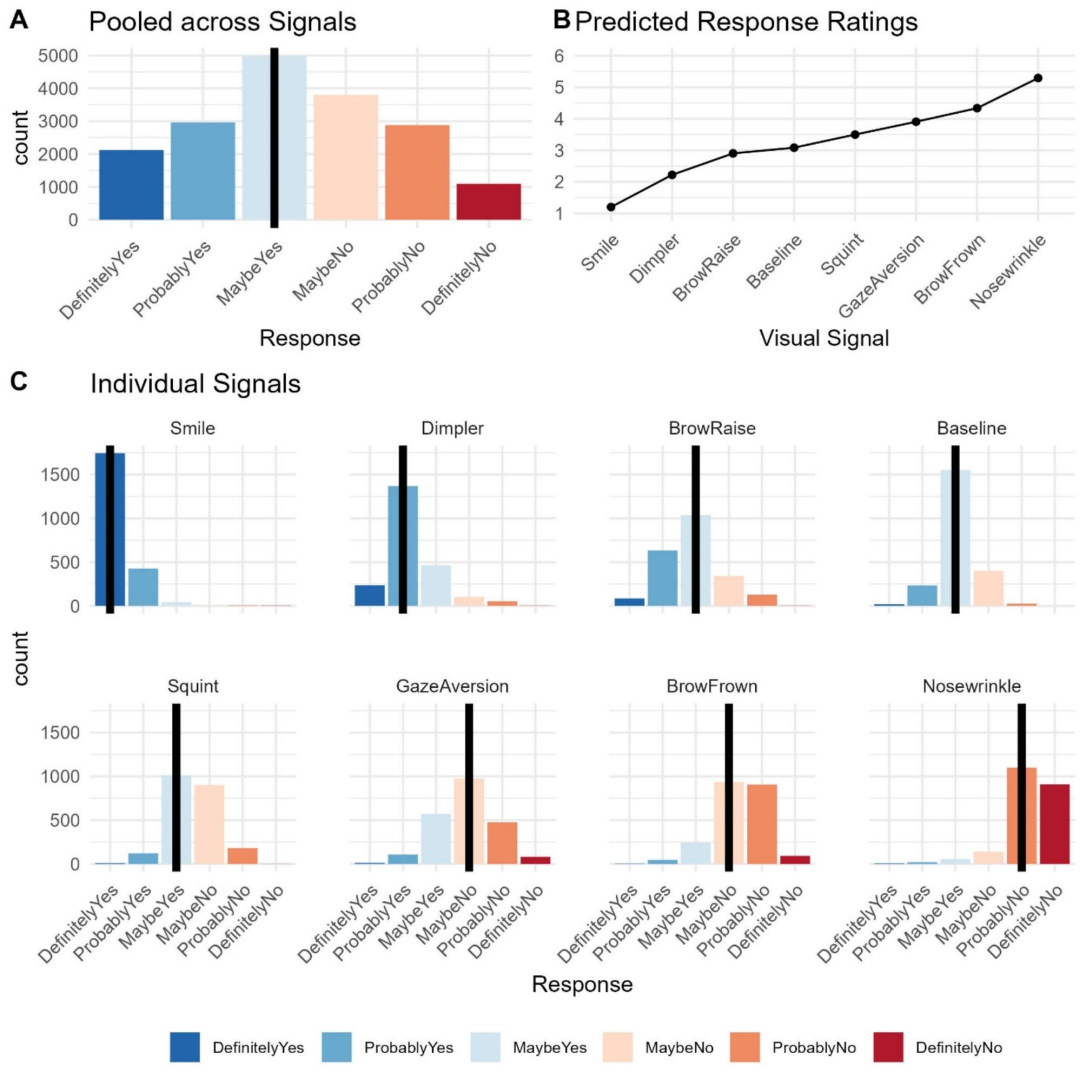
Distribution of response ratings. **A** Response ratings pooled across all facial signals. **B** Model estimated response ratings, where 1 corresponds to “DefinitelyYes”, and 6 to “DefinitelyNo”. Note that 95% confidence intervals are very small, and thus not visualized here (see Table 2 for values). **C** Response ratings for each individual signal. The median is highlighted by the vertical black bars in A and C.

The overall median response across all questions and visual signals was maybe yes (Fig. 2a). This was also the case for 190 items. Only 1 item received a median response rating of probably yes, 2 items a median rating between maybe yes and maybe no, and 39 items a median rating of maybe no.

Model comparisons revealed a significantly improved model fit for the cumulative link mixed-effects model including visual signal as a fixed effect (LR(7) = 23088, *p* < 0.001), confirming that visual signals are associated with differences in response ratings (Table 2). Pairwise comparisons revealed that response ratings were different for each visual signal compared to the baseline condition, and compared to the other visual signals (Supplementary Table S1, Fig. 2b, c). Specifically, smiles, brow raises, and dimplers were associated with more positive responses, while gaze aversions, brow frowns, nose wrinkles and squints were associated with more negative responses compared to the baseline condition. The response ratings differed across all visual stimuli: Smiles received the most positive response ratings, with a median rating of “definitely yes” (mean.class = 1.2, CI = 1.17–1.23), followed by dimplers with a median rating of “probably yes” (mean.class = 2.22, CI = 2.16–2.28), and brow raises with a median rating of “maybe yes” (mean.class = 2.90, CI = 2.84–2.97). The baseline condition of straight gaze also had a median rating of “maybe yes” (mean.class = 3.08, CI = 3.02–3.15), but the overall distribution of ratings was less positive than for brow raises. Squints were the most ambiguous signal, with a median rating of “maybe yes” (mean.class = 3.50, CI = 3.43–3.57), but overall 51% positive ratings, and 49% negative ratings. Next came brow frowns, with a median rating of “maybe no” (mean.class = 4.43, CI = 4.26–4.41), followed by nose wrinkles, which had the most negative response ratings with a median rating of “probably no” (mean.class = 5.29, CI = 5.23–5.35).

Supplementary analyses revealed that EQ score influenced the ratings of some visual signals, with higher EQ associated with more positive ratings for brow raises and more negative ratings for brow frowns (see Supplementary Materials for further details).

**Table 2:** Results of cumulative link mixed-effects Model.

Model comparisons							
	Fixed effects			Random effects		AIC	LogLik
Model 0	1			(1|Participant)+(1|Item)		60,322	-30,154
Model 1	1 + Signal			(1|Participant)+(1|Item)		37,249	-18,610
Model 1 has a significantly improved model fit compared to the null model (LRstat = 23088, *p* < 0.001)							
*Estimated Marginal Means Model 1*							
Signal		Emmean	Std. error		95% Confidence Interval		
Smile		1.20	0.0164		1.17	1.23	
Dimpler		2.22	0.0308		2.16	2.28	
Brow Raise		2.90	0.0335		2.84	2.97	
Baseline		3.08	0.0335		3.02	3.15	
Squint		3.50	0.0369		3.42	3.57	
Gaze Aversion		3.91	0.0383		3.83	3.98	
Brow Frown		4.34	0.0372		4.26	4.41	
Nosewrinkle		5.29	0.0299		5.23	5.35	

Estimates are based on the setting mode = mean.class in emmeans function, which returns the means of the ordinal response as a numeric value from 1 to 6, where 1 corresponds to “definitely yes” and 6 to “definitely no”.

## Discussion

Using a multimodal overhearer paradigm based on virtually animated characters, we examined whether observers form expectations about an upcoming conversational response based on facial signals produced before the verbal response is given. The findings revealed that facial signals can significantly influence response expectation. In line with prior work^10^, gaze aversions were associated with more dispreferred responses compared to the baseline condition with straight gaze. We further demonstrated associations between a range of other facial signals and response preference, with smiles, dimplers and brow raises eliciting more positive response expectations, and squints, brow frowns and nose wrinkles more negative response expectations relative to baseline.

While evidence from a conversational corpus has previously linked gaze aversions to dispreferred responses^10^, this is the first study to provide experimental evidence that visual signals produced prior to a verbal response shape observers’ expectations about the type of the upcoming response. Additionally, we provide the first evidence of an association of a range of facial signals typical for everyday face-to-face conversation with the response expectations that they elicit.

As hypothesized, smiles were associated with the most positive responses, with a median response rating of “definitely yes”, while nose wrinkles were associated with the most negative responses, with a median response rating of “probably no”. Due to their association with the “not face” we expected brow frowns, nose wrinkles and dimplers to be associated with more negative responses^31^. While this expectation was met for brow frowns and nose wrinkles, dimplers were in fact associated with more positive responses relative to baseline. One explanation for this might be that the compositionality of multiple signals influences how they are perceived, compared to signals produced in isolation^34,43^. In the original report of the ‘not-face’ by Benitez-Quiroz and colleagues^31^, there is no report on the frequency of individual facial signals or different combinations of signals, however the figures illustrating the ‘not-face’ all consist of multiple facial signals. On its own, the dimpler is visually similar to a one-sided smile, which is in line with its positive association in our data (but weaker than that for full smiles). Squints received the most ambiguous ratings, with 51% positive ratings and 49% negative ratings. While this is an overall more negative association compared to baseline, this observation suggests that squints might be associated with “thinking”^35^, suggesting that the addressee is uncertain and possibly contemplating their options. Interesting is also that straight gaze towards the speaker—a default orientation in conversation, especially when listening to questions^30,33^, which in itself is not affiliative as such—was associated with an overall positive response expectation. This seems to reflect interlocutors’ general pro-social orientation and preference for affiliative, face-preserving actions^44^, and the bias in conversational expectations this creates.

Additional exploratory analyses revealed that interindividual variations in social predispositions (EQ) may modulate the sensitivity to conversational visual signals and how they are used, to predict the nature of upcoming responses in conversation. Several clinical profiles may be characterized by differences in empathy as measured by the EQ. For example, autism^40^ and depression^45,46^ are both associated with lower EQ scores compared to neurotypical groups. These groups have also shown impacted facial expression recognition^47–49^. While these studies of facial expression recognition are often limited to studies of emotional expressions, it has been argued that communicative facial signals have evolved from facial displays of emotion^31,50^. It is therefore possible that impaired emotional facial recognition would carry over to the interpretation of conversational facial signals. For example, in the context of the current experiment, participants may predict the upcoming response by ascribing a particular emotional stance reflective of the upcoming verbal response to the avatar based on their facial expression.

This also points to the need for more interactions between the fields of language and emotion^51^, as facial signals may be attributed different functions depending on social, linguistic, and emotional context. For instance, the dimpler has previously been interpreted as a signal of contempt^52^, and is considered a component of the ‘not-face’^31^, but was associated with an overall positive response in our data, suggesting that the visual signal may be interpreted differently depending on dialogical and emotional context or the presence of other visual signals^43^. Another example illustrating contextual variation are smiles, which are typically associated with positive valence in the context of emotion, but depending on conversational context and tone may be interpreted sarcastically^53^. To further study on how precisely communicative facial signals are used to form predictions in social interaction, and how interindividual differences in social predispositions impact these capabilities, future studies may manipulate the visual, social and emotional context of the stimuli in their experimental designs.

In this experiment, we have demonstrated that visual signals produced by an addressee prior to giving a verbal response as next speaker can allow interlocutors to form expectations about the nature of the upcoming response, thus adding to the growing body of literature on the predictive power of visual bodily signals^18,21,26,27^. Facial signals with predictive power are of considerable interactional significance, since by anticipating a dispreferred response, they may allow a speaker to redesign their turn to allow for a more positive/affiliative response instead. Such behavior has been reported by Kendrick and Holler^10^, in their qualitative analysis of addressee gaze and response preference. Visual bodily signals may thus do much more than simply contribute semantic or pragmatic information to what is being said; through their predictive power, they may critically influence the course of conversational interactions. This study has provided the first experimental evidence for this possibility. The present findings thus provide the foundation for future studies designed to tap online interaction and linguistic cognition in multimodal, face-to-face conversations and the social processes that shape them.

## Electronic supplementary material

Below is the link to the electronic supplementary material.

Supplementary Material 1.

## Data Availability

Data and code are available at https://osf.io/5ehcm/.
