# Supplementary material for: Facial signals shape predictions about the nature of upcoming conversational responses
Source: Sci Rep. 2025 Jan 9;15:1381. doi: 10.1038/s41598-025-85192-y (PMC11711643; doi:10.1038/s41598-025-85192-y)
Supplement: Supplementary file 1 — Supplementary Material 1. [file 41598_2025_85192_MOESM1_ESM.docx]

**Supplementary Materials**

**Facial signals shape predictions about the nature of upcoming conversational responses**

Alexandra K Emmendorfer*^1,2^, Judith Holler^1,2^

1. Donders Institute for Brain, Cognition & Behaviour, Radboud University, Nijmegen, The Netherlands
2. Max Planck Institute for Psycholinguistics, Nijmegen, The Netherlands

*Corresponding author: alexandra.emmendorfer@mpi.nl

**Supplementary Figure S1**

*Participant excluded based on performance*

**
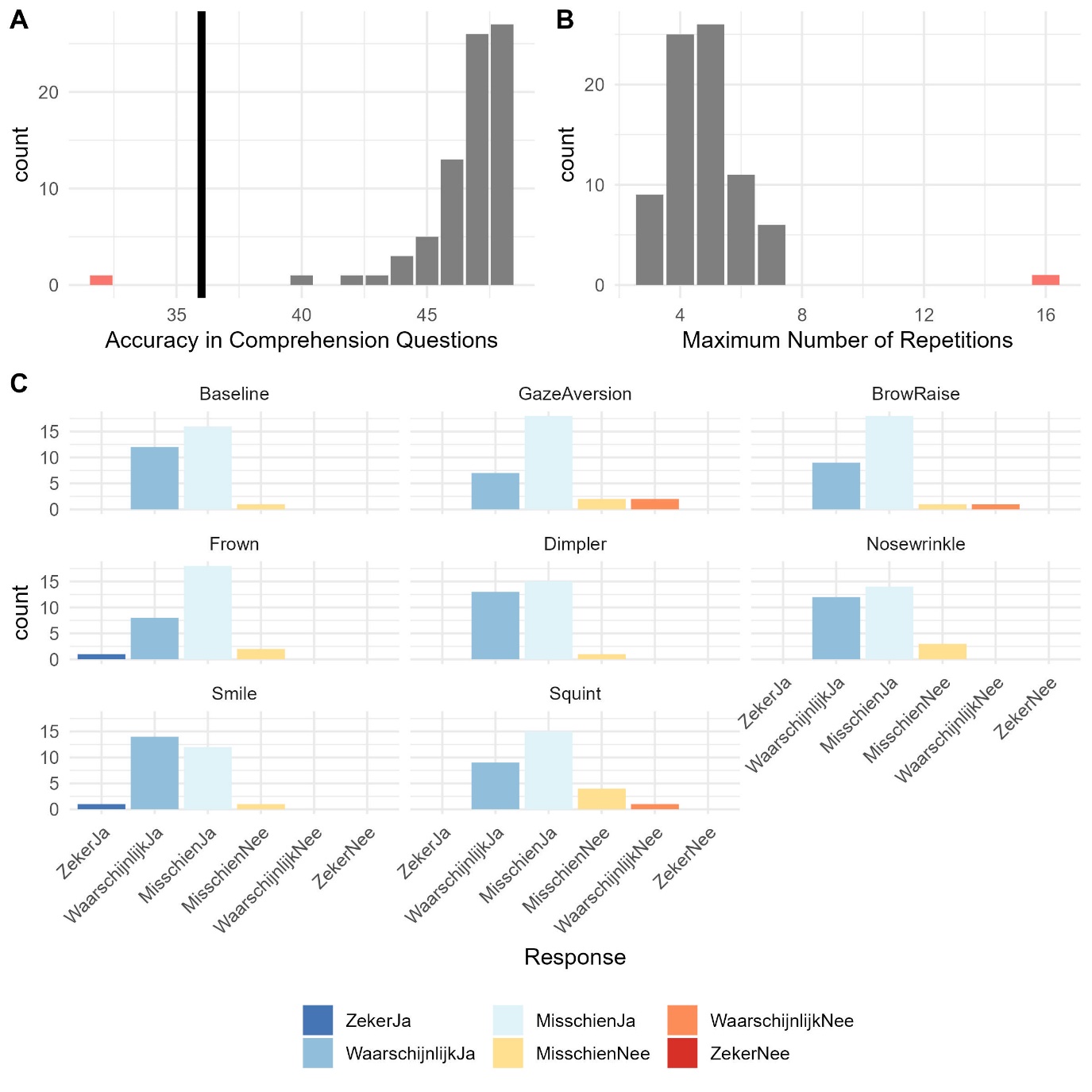
**

*Note.* (A) Number of comprehension questions answered correctly per participant. Black vertical line indicates the preregistered threshold (36 questions, or 75% accuracy) for deciding on inclusion based on response behavior. One participant (indicated in red) with 32 correct responses (66.7% accuracy) fell below this threshold. (B) Maximum number of sequentially repeated ratings per participant. The participant identified in A showed an unusual number of repetitions compared to other participants. This participant’s response rating per visual signal is shown in (C).

**Supplementary Figure S2**

*Questionnaire scores per participant*
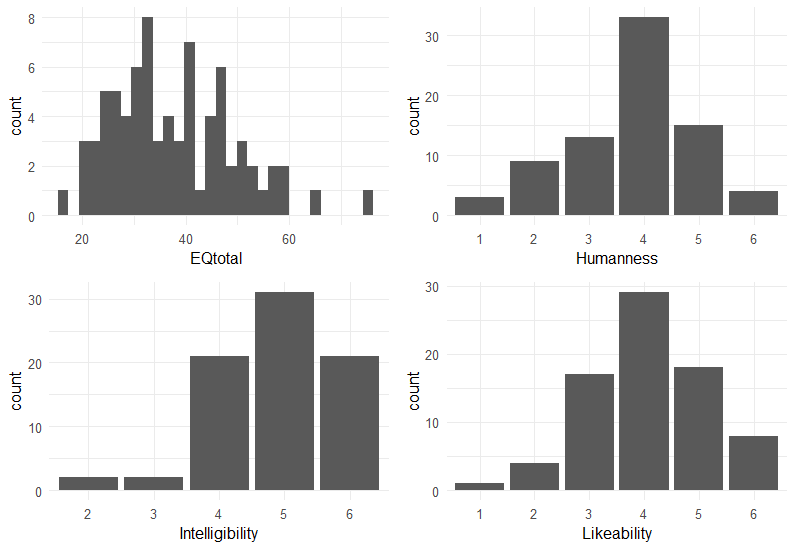


*Supplementary analyses – Empathy Quotient*

To assess whether individual differences in Empathy Quotient were associated with the effect of visual signals on response ratings, we fit a model with the interaction between visual signal and normalized EQ score as fixed effects, and random intercepts for participant and item as main effect. This model was compared to the previously fit model with the anova function, revealing a significantly improved model fit for the main model (Supplementary Table S4). The ratings for brow raises and brow frowns were significantly influenced by EQ score, where higher EQ scores were associated with more positive ratings for brow raises (slope = -0.09360, 95% CI = -1.553 - -0.0319), but more negative ratings for brow frowns (slope = 0.10698, 95% CI = 0.0383 – 0.1757). These effects may have been driven by one outlying EQ score (>3 SD greater than mean), thus the analysis was repeated with this participant removed. Both brow raise (slope = -0.07401, 95% CI = -0.14095 - -0.00708) and brow frown (slope = 0.08234, 95% CI = 0.00773 – 0.15695) effects remained. The response ratings by normalized EQ score are plotted in Supplementary Figure S3.

**Supplementary Figure S3**

*Response ratings by normalized EQ score*

*
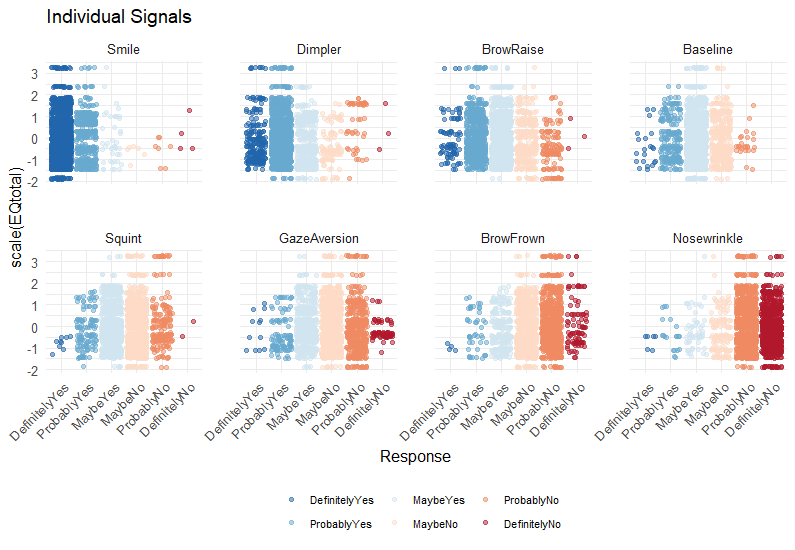
*

*Note.* Response ratings for each individual signal plotted against normalized EQ score.

**Supplementary Table S1**

*Pairwise comparisons.*

|  | Dimpler | BrowRaise | Baseline | Squint | GazeAversion | BrowFrown | Nosewrinkle |
| --- | --- | --- | --- | --- | --- | --- | --- |
| Smile | -1.020 (0.0217) | -1.700 (0.0241) | -1.881 (0.0238) | -2.297 (0.0268) | -2.704 (0.0282) | -3.134 (0.0273) | -4.090 (0.0210) |
| Dimpler |  | -0.681 (0.0210) | -0.862 (0.0204) | -1.277 (0.0220) | -1.684  (0.0230) | -2.115 (0.224) | -3.069 (0.0198) |
| BrowRaise |  |  | -0.181 (0.0211) | -0.596 (0.0224) | -1.003 (0.0233) | -1.434 (0.0228) | -2.388 (0.0210) |
| Baseline |  |  |  | -0.415 (0.0216) | -0.822 (0.0225) | -1.253 (0.0220) | -2.207 (0.0203) |
| Squint |  |  |  |  | -0.407 (0.0231) | -0.838 (0.0228) | -1.792 (0.0221) |
| GazeAversion |  |  |  |  |  | -0.431 (0.0235) | -1.385 (0.0231) |
| BrowFrown |  |  |  |  |  |  | -0.954 (0.0225) |

*Note.* Values indicate model estimated difference between conditions (row – column) and the standard error in parentheses. Negative values indicate that the condition in the row was associated with more positive responses than the condition in the column. All contrasts were significant with p<0.0001 after Bonferroni correction.`

| **Supplementary Table S2:**  *Results of Cumulative Link Mixed-Effects Model for n = 80* | | | | | | | |
| --- | --- | --- | --- | --- | --- | --- | --- |
| *Model Comparisons* | | | | | | | |
|  | | Fixed effects | | Random effects | | AIC | LogLik |
| Model 0 | | 1 | | (1\|Participant)+(1\|Item) | | 62415 | -31200 |
| Model 1 | | 1 + Signal | | (1\|Participant)+(1\|Item) | | 39080 | -19526 |
| Model 1 has a significantly improved model fit compared to the null model (LRstat = 23349, p<0.001) | | | | | | | |
|  | | | | | | | |
| *Estimated Marginal Means Model 1* | | | | | | | |
| Signal | mean | | Std. error | | 95% Confidence Interval | |  |
| Smile | 1.21 | | 0.0166 | | 1.18 | 1.24 |  |
| Dimpler | 2.23 | | 0.0306 | | 2.17 | 2.29 |  |
| Brow Raise | 2.91 | | 0.0332 | | 2.84 | 2.97 |  |
| Baseline | 3.08 | | 0.0332 | | 3.01 | 3.14 |  |
| Squint | 3.49 | | 0.0365 | | 3.42 | 3.56 |  |
| Gaze Aversion | 3.87 | | 0.0382 | | 3.80 | 3.94 |  |
| Brow Frown | 4.31 | | 0.0374 | | 4.23 | 4.38 |  |
| Nosewrinkle | 5.26 | | 0.0302 | | 5.20 | 5.32 |  |

*Note.* This analysis includes 3 participants that were excluded from the main analysis to highlight the robustness of the findings.

**Supplementary Table S3**

*Pairwise comparisons for n = 80.*

|  | Dimpler | BrowRaise | Baseline | Squint | GazeAversion | BrowFrown | Nosewrinkle |
| --- | --- | --- | --- | --- | --- | --- | --- |
| Smile | -1.017  (0.0213) | -1.698  (0.0236) | -1.867  (0.0233) | -2.276  (0.0263) | -2.659  (0.0279) | -3.096  (0.0272) | -4.046  (0.0210) |
| Dimpler |  | -0.681  (0.0207) | -0.850  (0.0200) | -1.259  (0.0217) | -1.641  (0.0228) | -2.079  (0.0224) | -3.029  (0.0196) |
| BrowRaise |  |  | -0.169  (0.0207) | -0.577  (0.0221) | -0.960  (0.0230) | -1.398  (0.0226) | -2.347  (0.0207) |
| Baseline |  |  |  | -0.408  (0.0213) | -0.791  (0.0223) | -1.229  (0.0219) | -2.178  (0.0201) |
| Squint |  |  |  |  | -0.383  (0.0229) | -0.820  (0.0226) | -1.770  (0.0218) |
| GazeAversion |  |  |  |  |  | -0.437  (0.0233) | -1.387  (0.0229) |
| BrowFrown |  |  |  |  |  |  | -0.950  (0.0224) |

*Note.* This analysis includes 3 participants that were excluded from the main analysis to highlight the robustness of the findings. Values indicate model estimated difference between conditions (row – column) and the standard error in parentheses. Negative values indicate that the condition in the row was associated with more positive responses than the condition in the column. All contrasts were significant with p<0.0001 after Bonferroni correction.

| **Supplementary Table S4:**  *Results of Cumulative Link Mixed-Effects Model including normalized EQ scores* | | | | | | | | | |
| --- | --- | --- | --- | --- | --- | --- | --- | --- | --- |
| *Model Comparisons* | | | | | | | | | |
|  | Fixed effects | | Random effects | | | AIC | LogLik | | |
| Model 1 | 1 + Signal | | (1\|Participant)+(1\|Item) | | | 37249 | -18610 | | |
| Model 2 | 1 + Signal*scale(EQ) | | (1\|Participant)+(1\|Item) | | | 37176 | -18566 | | |
| Model 2 has a significantly improved model fit compared to the null model (LRstat =89.064, p<0.001) | | | | | | | | | |
|  | | | | | | | | | |
| *Estimated Marginal Means & EQscaled Slopes Model 2* | | | | | | | | | |
| Signal | | Mean (95% CI) | | | Slope (95% CI) | | |  |  |
| Smile | | 1.20 (1.17 - 1.23) | | 0.00401 (-0.0328 – 0.1033) | | | | | |
| Dimpler | | 2.22 (2.16 - 2.28) | | -0.01999 (-0.0771 – 0.0371) | | | | | |
| Brow Raise | | 2.91 (2.84 - 2.97) | | -0.09360 (-1.553 - -0.0319) | | | | | |
| Baseline | | 3.08 (3.02 - 3.15) | | -0.00602 (-0.0678 – 0.0558) | | | | | |
| Squint | | 3.50 (3.43 - 3.57) | | 0.03527 (-0.0328 – 0.1033) | | | | | |
| Gaze Aversion | | 3.91 (3.83 - 3.98) | | -0.00573 (-0.0760 – 0.0645) | | | | | |
| Brow Frown | | 4.34 (4.26 - 4.41) | | 0.10698 (0.0383 – 0.1757) | | | | | |
| Nosewrinkle | | 5.29 (5.23 - 5.35) | | 0.02316 (-0.0319 – 0.0783) | | | | |  |
